# Fresh water skin disease in dolphins: a case definition based on pathology and environmental factors in Australia

**DOI:** 10.1038/s41598-020-78858-2

**Published:** 2020-12-15

**Authors:** Pádraig J. Duignan, Nahiid S. Stephens, Kate Robb

**Affiliations:** 1 The Marine Mammal Center, 2000 Bunker Road, Sausalito, CA 94965 USA; 2 School of Veterinary Medicine, Murdoch University, 90 South Street, Murdoch, WA 6150 Australia; 3 Marine Mammal Foundation, Hampton East 3188, PO Box 2046, Victoria, Australia

**Keywords:** Ecology, Ocean sciences, Diseases, Pathogenesis, Risk factors, Signs and symptoms

## Abstract

A distinct ulcerative dermatitis known as “*freshwater skin disease*” is an emerging clinical and pathological presentation in coastal cetaceans worldwide. In Australia, two remarkably similar mortality events enabled the creation of a case definition based on pathology and environmental factors. The first affected a community of endemic *Tursiops australis* in the Gippsland Lakes, Victoria, while the second occurred among *T. aduncus* resident in the Swan-Canning River system, Western Australia. The common features of both events were (1) an abrupt and marked decrease in salinity (from > 30ppt to < 5ppt) due to rainfall in the catchments, with hypo-salinity persisting weeks to months, and (2) dermatitis characterized grossly by patchy skin pallor that progressed to variable circular or targetoid, often raised, and centrally ulcerated lesions covering up to 70% of the body surface. The affected skin was often colonized by a variety of fungal, bacterial and algal species that imparted variable yellow, green or orange discoloration. Histologic lesions consisted of epidermal hydropic change leading to vesiculation and erosion; alternately, or in addition, the formation of intra-epithelial pustules resulting in ulceration and hypodermal necrosis. Thus, the environmental factors and characteristic pathologic lesions, are necessary components of the case definition for freshwater skin disease.

## Introduction

Dermatopathology has long been used as a proxy for health in cetaceans with adverse environmental conditions assumed to cause physiological stress and impaired immunological function that can manifest as epidermal disease^1–14^ Given the obvious logistical challenge of assessing the health of free-ranging dolphins, their lack of hair or fur is fortunate in that the photographic catalogues widely used in cetacean ecology and ethology research, have found a novel use in recording the prevalence, severity and temporal trends in lesion appearance reducing the need for capture or restraint. The limitation of this technique of course is that the etiology of the lesions can remain elusive. Severe, multi-focal to confluent ulceration of the skin, often associated with overgrowth of velvety orange algal and fungal mats, was first reported in 2007 when lesions were fortuitously observed in a group of up to 40 common bottlenose dolphins (*Tursiops truncatus*) entrapped in Lake Pontchartrain, LA. The dolphins were thought to have entered the brackish lake, used as a flood spillway for the Mississippi River, following Hurricane Katrina in August 2005^13^. Regular surveys of these out-of-habitat dolphins were conducted seasonally over the subsequent three years and it was noted that skin lesions occurred on up to 100% of the animals photographed. Lesion severity varied seasonally, waxing and waning in known individuals as salinity declined or increased^13^. Over the study period, numerous animals stranded but there was no histopathologic investigation of the dermatitis. A similar scenario ensued in August 2017 when Hurricane Harvey inundated Galveston Bay, TX, with rainfall decreasing salinity in the bay from 14ppt to < 1ppt^14^. Long-term photo-identification studies of the resident bottlenose dolphins provided an ideal opportunity to document how dolphins evacuated the hyposaline waters of the upper bay and changes in the extent and severity of grossly-visible skin lesions (pallor and ulceration) before, during and after the hurricane. While these were the first studies to associate gross skin lesions with prolonged exposure to hypo-saline water, an earlier study by Wilson et al.^2^, also based on photographs of free-ranging bottlenose dolphins, made the association between dermatopathology and environmental factors. In this survey of ten separate dolphin communities from Europe, North America and New Zealand, representing a range of latitudes, temperature and salinity regimes, and contaminant burdens, the authors found significant associations between lesion prevalence and severity with both salinity and water temperature. By contrast, there was no association between lesion prevalence or severity with a range of anthropogenic chemicals previously reported for dolphin blubber from strandings in these regions^2^.

Bottlenose dolphins inhabit coastal and estuarine ecosystems from the tropics to high temperate latitudes around the globe that are subject to seasonal, annual or stochastic change through natural and anthropogenic influences. An unpredictable environment can present many challenges to their health. For dolphins of the Gippsland Lakes, Victoria, and the Swan-Canning River system, Western Australia, these stressors include the physiological stress of inhabiting a highly-variable environment; the physical trauma of becoming entangled in fishing gear; the immunological challenge of being exposed to natural pathogens and to industrial or agricultural contaminants; the disturbance caused by increasing vessel traffic and anthropogenic noise; and the environmental stress of living in an ecosystem adjacent to a major metropolitan area or that is surrounded by a large agricultural catchment. Australia is noted for diversity in its coastal marine mammals, including a recently-proposed new species *T. australis,* known locally as the Burrunan dolphin, with genetically isolated populations in the south east of the continent, including the Gippsland Lakes^15–21^. While the species classification is contested by some^22,23^, a large body of evidence supports validating the Burrunan as a separate species from the common and Indo-Pacific bottlenose dolphins (*T. truncatus* and *T. aduncus*, respectively), using mtDNA regions^24^, concatenated mtDNA/nuDNA sequences^25^, the mitogenome^26–29^, and more recently, the time-calibrated molecular phylogeny of Certiodacyla^30^.

Australia, however, is also known as a region that experiences extreme climatic and rainfall variation; where a clear link between environmental forces and coastal marine mammal mortality has been demonstrated, at least for the tropical north^31^. Climatic variation along Australian coastlines is largely dominated by the El Niño-Southern Oscillation (ENSO) phenomenon^32^, and often alternates between drought- and flood-dominated periods. However, there are multiple drivers interacting in a complex interplay to influence climate. The west coast in particular is strongly influenced by the Indian Ocean Dipole (IOD), which is capable of overriding the ENSO’s influence on cool season rainfall across Australia’s eastern seaboard^33^. The southern west coast of Australia, where one of our study’s populations is situated, has been classified as one of 24 global warming hot spots and has already seen devastating impacts in regions famous for their marine biodiversity^34^. Since the early 1900s, air and sea surface temperatures in the region have significantly warmed and are predicted to continue to increase, while sea levels are predicted to rise and pH fall^35^. Higher oceanic temperatures are projected, particularly for south-eastern Australia where the second study population is located. The East Australia Current is projected to transport greater volumes of water southward, whereas the Leeuwin Current on the west coast may weaken^36^. Projections suggest that, on land, air temperatures will rise and rainfall will decline across much of Australia in coming decades; the combination of these drivers will likely result in overall reduced runoff and therefore reduced stream flow and lake storage^36^. However, present climate models are particularly limited with regard to coastal and freshwater systems, making them challenging to use for biological-impact and adaptation studies^36^. Therefore, exactly how warming temperatures will interact with the complex interplay of drivers as outlined above is uncertain, but precipitation extremes and the frequency of severe weather events such as floods, storms and cyclones are expected to increase into the future^35^. Even if the overall trend is towards reduced runoff, stochastic extremes are likely to result in more devastating flood events for estuaries, lagoons and coastal marshes, and/or more rapid fluctuations in conditions. The impact that these environmental changes will have on coastal dolphin communities remains to be determined but continued monitoring for skin lesions is likely to be a useful and non-invasive method for researchers.

The purpose of this study is to provide a case definition for fresh-water skin disease in bottlenose dolphins based on gross and histologic lesion presentation and environmental associations. While the case scenarios are from dolphins in Australian waters, the case definition is broadly applicable globally to any cetacean species inhabiting similar coastal or estuarine habitats.

## Methods and results

### Gippsland Lakes, Victoria, mortality event October–November, 2007

**Figure 1:**
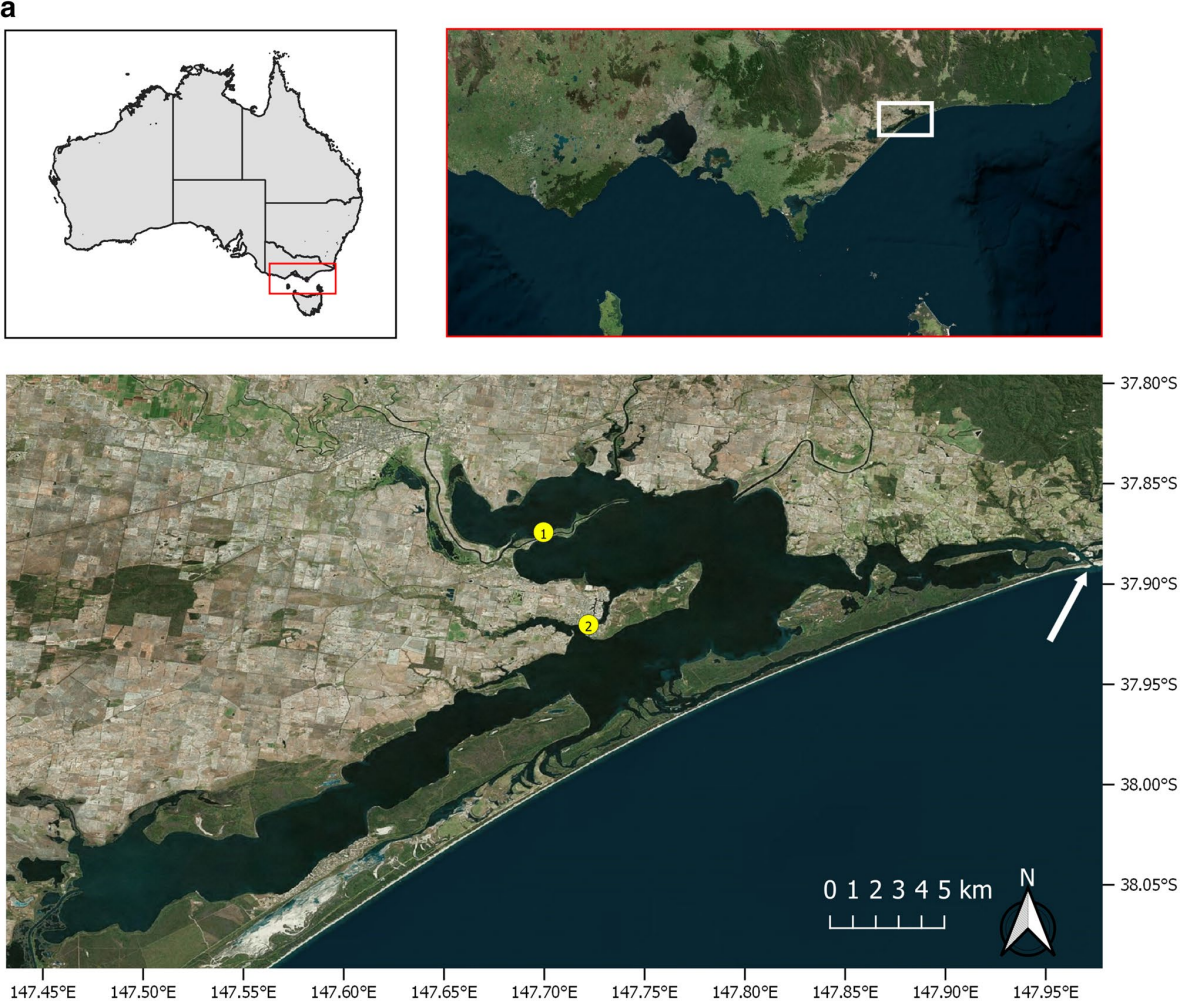

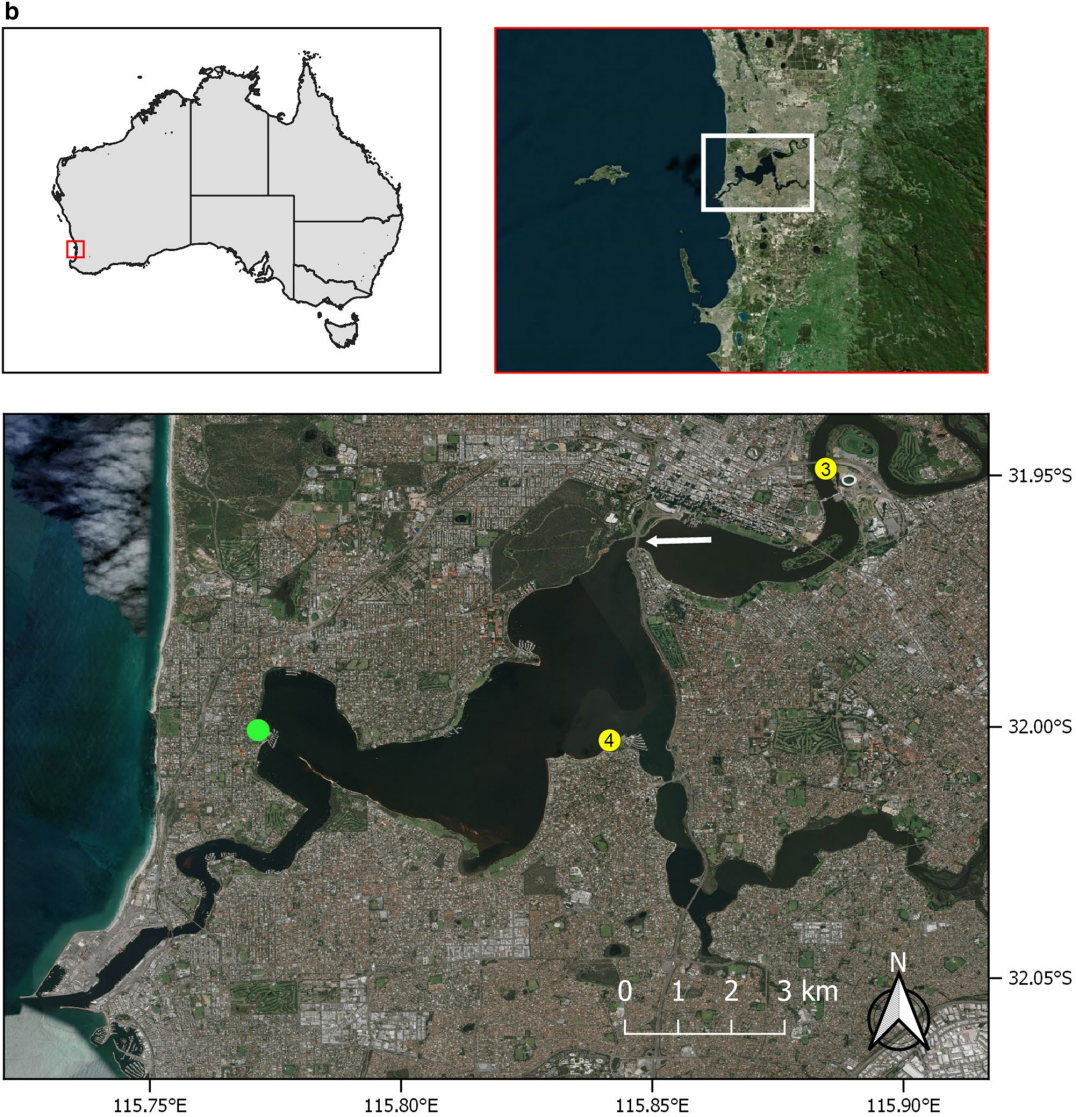
(**a**) The red and white rectangles show the location of the Gippsland Lakes in eastern Victoria, Australia (upper maps). The lower map shows the stranding locations (yellow dots) on Lake King for the adult female *Tursiops australis* (Case 1) found dead 29th Oct. 2007, carcass code 2 (fresh). The adult male *T. australis* (Case 2) was found 1st Nov. 2007, carcass code 2. The arrow indicates Lakes Entrance where the lakes opens to the Tasman Sea. Maps were produced using QGIS v3.10.11 (with Bing aerial insert). QGIS Geographic Information System, Open Source Geospatial Foundation Project. http://qgis.org. (**b**) The red and white rectangles show the location of the Swan-Canning river system in Western Australia. The lower map shows the stranding locations (yellow dots) for two *Tursiops aduncus* found dead with severe ulcerative dermatitis and the location of a third dolphin (green dot) not retrieved for necropsy. The white arrow indicates The Narrows Bridge. (3) Adult female found dead 17 Sept. 2009 (Case 3). Carcass code 2 (fresh). (4) Adult female observed agonal 25 Oct. 2009 (Case 4). Carcass code 2 (fresh). Maps were produced as for (**a**).

The Gippsland Lakes (600km^2^) in eastern Victoria, south east Australia comprise a series of brackish lakes, lagoons and marshes fed by seven rivers and only separated from the Tasman Sea by a sandbar, Ninety Mile Beach (Fig. 1a)^37^. They open to the sea at one location, Lakes Entrance (37^o^ 52′ 0″ S, 147^o^ 57′ 0″ E), an 80 m wide artificially created and maintained opening^37^. The rivers drain an extensive catchment area that is predominantly agricultural land. Listed as a Wetland of International Importance under the Ramsar Convention, the area has great ecological significance, not least as the home range for approximately 65 endemic and endangered Burrunan dolphins, *T. australis*^18,19^. Between late October and early November 2007, three adult residents were found dead over a 10-day period, two of which had extensive epidermal ulceration. The first case (Case 1), an adult female, was found in the water at Jones Bay on the Mitchell River on 29th October. The second (Case 2), an adult male, stranded at Paynesville on 1st November and based on dental attrition, it was of advanced years. Both dolphins were recently dead (code 2) and suitable for necropsy and histopathology^38^. Dolphin necropsies and tissue sampling in Victoria were conducted under the Wildlife Act 1975 Research Permit #10003250, issued by Victorian Department of Environment and Primary Industries (DEPI; Victorian State Government) and was approved by the Biological Sciences Animal Ethics Committee (Monash University) BSCI/2008/21. The laboratory work was funded by contributions from the West Gippsland Catchment Management Authority (http://www.wgcma.vic.gov.au/), Gippsland Lakes Task Force and Coast Action/Coastcare (http://www.coastcare.com.au/).

Tissue sampling was comprehensive following standard protocols^39^. Multiple skin lesions were selected for sampling that ranged in severity from mild skin pallor to deep ulceration. All lesions were sampled at the junction of relatively normal skin and the lesion and fixed in 10% neutral buffered formalin. Skin samples were additionally stored chilled or frozen for bacterial and fungal culture, liver and kidney were stored frozen for heavy metal analysis and blubber (hypodermis) for organochlorine contaminants. Fixed skin samples were embedded in paraffin, sectioned at 5-μm thickness, stained histochemically with hematoxylin and eosin (H&E), gram, giemsa, acid fast bacteria (AFB) stain, periodic acid Schiff (PAS) and Grocott -Gomori’s methenamine silver (GMS) stains^40^. For case 1, there were no gross abnormalities noted for the internal organs and no tissue samples were processed for histopathology. For case 2, lungs, liver, spleen, kidneys, stomach, intestine and colon were examined microscopically in addition to multiple skin samples. The only significant finding for the internal organs was mild chronic glomerular sclerosis consistent with the dolphin’s age.

Through November 2007, boat-based photography surveys across the Gippsland Lakes carried out to assess the status of the remaining resident dolphins, showed approximately 40% of 40 resident individuals photographed had a range of epidermal lesions with varying degrees of severity. Lesions were reported on individual dolphins into February 2008. An earlier series of surveys, conducted in March and June of 2007, prior to the flood event, did not record any significant skin lesions (Robb, unpublished data).

### Swan-Canning Riverpark, Western Australia, mortalities in 2009

The Swan-Canning Riverpark is an extensive and complex waterway comprising, from north to south, the Inner Harbor adjacent to the city of Perth, WA (31^o^ 57′ 8″ S, 115^o^ 51′ 32″ E), the confluence of the Swan and Canning rivers, a series of broad basins called the Middle Reaches, and the Swan River down to the Indian Ocean at the Port of Fremantle (32^o^ 03′ 25″ S, 115^o^ 44′ 38″ E) (Fig. 1b). Based on photographic surveys conducted between 2001 and 2003, 35 individual Indo-Pacific bottlenose dolphins (*T. aduncus*) were regularly observed in the Riverpark with 18 of these regarded to be a resident community while the remaining dolphins were infrequent visitors^41,42^. Two distinct dolphin mortality events occurred in the Riverpark in 2009. The first event involved three younger animals (calf, juvenile and subadult) that were found dead in June prior to the winter rains. One carcass was too decomposed for further diagnostics but the other two died from complications of cetacean morbillivirus (CeMV) infection. Only one animal had skin pathology but that was associated with fishing line entanglement^43^. The second mortality event occurred in September and October and by contrast, affected only adult dolphins. This event occurred after a prolonged period of rain that transformed the habitat from marine to freshwater. Two of the affected dolphins were female (Cases 3 and 4) and they were in a good state of preservation for necropsy (code 2) but an adult male was in advanced decomposition and the carcass was not recovered.

The mortality investigtion was conducted under Animal Ethics Committee approvals DEC AEC 2005/01 and 2008/08 and Scientific Purposes Licenses SF000007, SC000619, SC000941 and SC001255 issued by the Department of Biodiversity, Conservation, and Attractions (formerly Swan River Trust), Western Australia. As the relevant governmental regulatory body, this department granted permission for access to and use of each cadaver for research purposes. Necropsies were carried out under Murdoch University’s scientific license to use animal cadavers for research purposes (Animal Ethics Committee, Murdoch University). The laboratrory work was funded by the Department of Biodiversity, Conservation, and Attractions (formerly Swan River Trust), Western Australia (RSP10MUR02). Necropsy and tissues sampling was conducted on the adult female dolphins as described above. In addition to the skin lesions, case 3 had moderate edematous enlargement of her peripheral lymph nodes (consistent with the severity of the dermatitis), mild multifocal gastric and proximal duodenal ulceration (associated with nematodes and also consistent with chronic stress), and hepatic biliary hyperplasia associated with trematode infestation. Case 4 was in poor body condition with generalized atrophy of the hypodermis (blubber layer) and skeletal muscle (malnutrition), chronic ulcerative esophagitis associated with an embedded fishing hook, moderate peripheral lymphadenopathy (chronic dermatitis), mild multifocal gastric ulceration (nematode infestation and chronic stress), diffuse acute pulmonary edema (agonal) and mild focal suppurative pneumonia (bacterial infection), and mild chronic myocardial interstitial fibrosis (old age).

Retrospective review of dolphin stranding reports for the Riverpark found an adult lactating female in good body condition and good state of preservation in November 2007. At necropsy she had multifocal circular skin lesions with locally extensive ulceration on the head caudal to the blowhole. On 27th October 2003, a dolphin of unspecified age or sex was stranded at Ascot in the Riverpark. It was not recovered or necropsied but photographs archived by the Swan River Trust (SRT) show that the fresh carcass had dorsal and lateral skin ulcers similar to those described for the case series animals.

### Clinical observations

The dolphins (Cases 1 & 2) found dead in the Gippsland Lakes in 2007 were not seen alive. However, the two adult female dolphins from the Swan-Canning Riverpark in 2009 were observed alive for days or weeks before death. The first (Case 3) lingered around a particular area of the Swan River for a few days and based on her reluctance to leave, was suspected to be ill and was found dead shortly after on 17th September 2009. The second dolphin (Case 4) was sighted three weeks prior to death by staff of the SRT who reported that she had skin lesions. On 25th October 2009 a dolphin (presumably the same individual) was seen in the Applecross region of the Swan River and was reported early in the afternoon to be swimming slowly and erratically, appearing distressed. By mid to late afternoon she had moved around Point Heathcote to Waylen Bay where she was observed by the Marine Police, who were subsequently joined by Murdoch University dolphin researchers. By then, the dolphin was very weak, swimming in the shallows leaning and circling to the left, while remaining at the surface. The decision was made to euthanase her, however shortly thereafter (< 20 min) she beached herself in the shallows on her left side and died approximately 5 min later following agonal spasms. The proximate cause of death may have been asphyxiation as her blowhole was submerged.

### Gross necropsy findings

**Figure 2:**
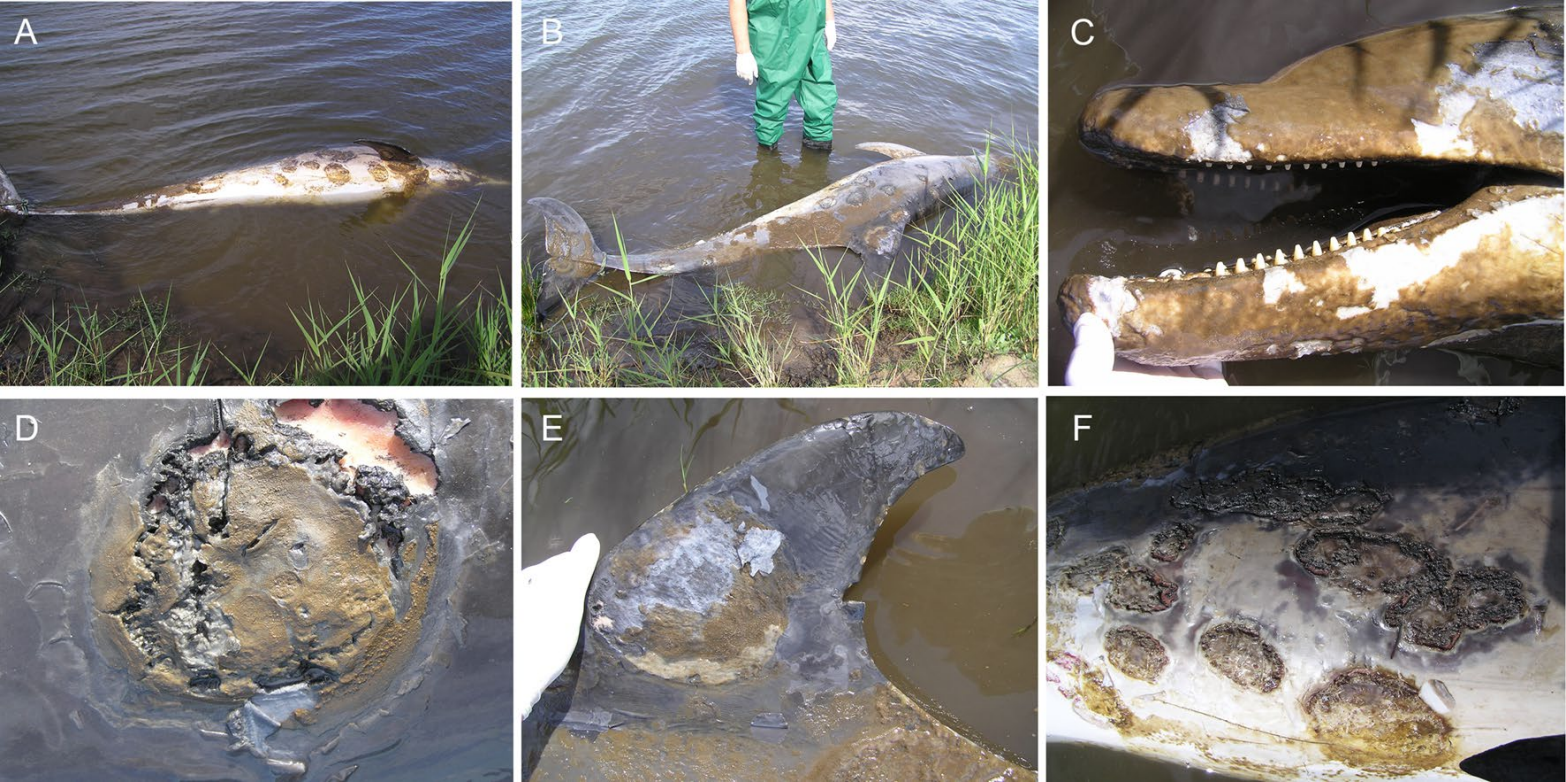
Adult female *T. australis* (case 1) found dead with severe ulcerative dermatitis at Jones Bay, Lake King North, near the mouth of the Mitchell River, 29th October, 2007. Discrete circular to confluent areas of epidermal thickening and ulceration, with overgrowth of algal mats, are randomly distributd on both sides of the body from the rostrum to tail flukes (**A**,**B**). Locally extensive algal mats on the head, melon, rostrum and mandibles (**C**). Closer view of a large raised, targetoid, focally ulcerated, circular plaque on the thorax with a thickened surface discolored yellow–brown (**D**). The dorsal fin has a large circular area of erosion and ulceration extensively overgrown by algae and similar dense algal mats are apparent on the dorsal trunk (**E**). The abdomen and thorax have focally extensive ulcers and plaques with overgrowth of algal mats (**F**).

**Figure 3:**
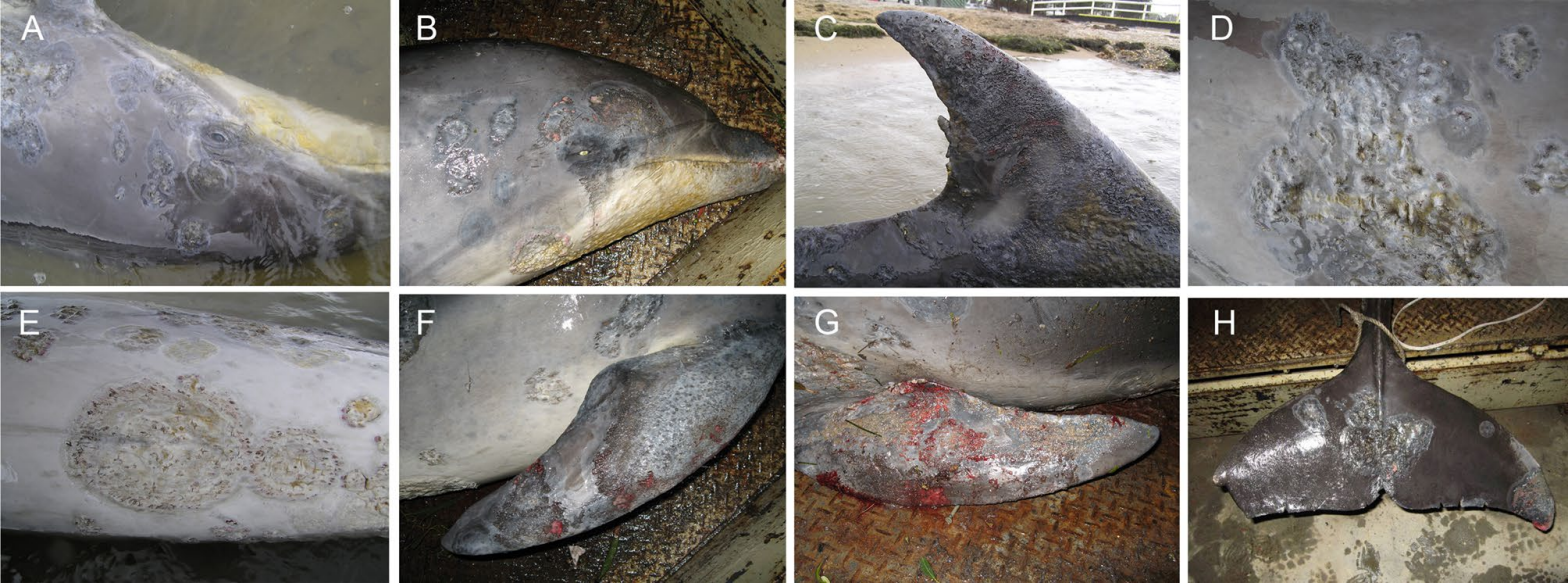
Adult male *T. australis* (case 2) found dead near Paynesville on Lake King North with extensive epidermal ulceration and discoloration on 1st November, 2007. The lesion morphology and distribution on the head (**A**,**B**), dorsal fin (**C**), flank (**D**), abdomen (**E**), right pectoral flipper (**F**), left pectoral flipper (**G**) and tail flukes (**H**) are similar to those for the adult female found in the same general area (Fig. 2).

**Figure 4:**
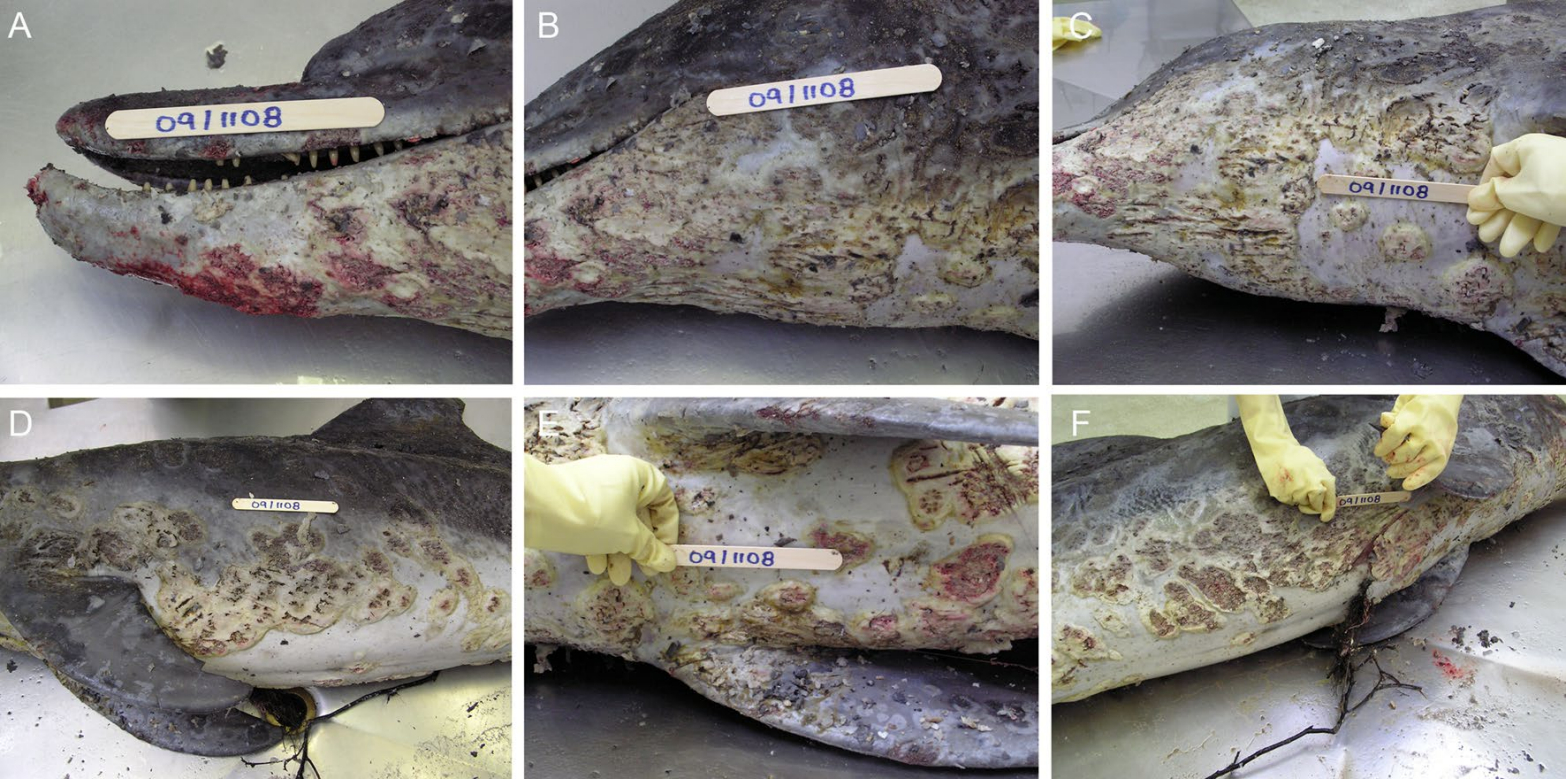
Adult female *T. aduncus* (case 4) that died in the Swan-Canning Riverpark on 25th October, 2009. There are extensive circular or targetoid, discrete to confluent plaques and ulcers affecting the mandible and rostrum (**A**), face and neck (**B**), ventral neck and thorax (**C**), the left left flank and pectoral flipper (**D**), sternum and plantar pectoral flippers (**E**), right abdomen and thorax (**F**).

Dermatopathology was the consistent finding for all four cases and based on the uniformity in presentation between cases, the following is a composite morphologic lesion description. Skin lesions varied in severity from localized patchy areas of pallor or discoloration to elevated pale vesicles, multifocal to coalescing, irregularly shaped, raised, umbilicated pale-grey to yellow-tinged plaques that frequently had central erosion or ulceration and necrosis often conferring a targetoid appearance (Figs. 2A–F, 3A–H, 4A–F). The skin in these areas was swollen, edematous and macerated, and readily sloughed. With gentle pressure, many of these lesions exuded a small volume of blood-tinged turbid fluid (serosanguineous exudate). On cut surface, the lesions were confined to the epidermis and occasionally the superficial dermis or hypodermis (blubber). The extent of surface area affected was variable up to approximately 70% with the distribution extending from the rostrum and head along the neck, thorax, abdomen, peduncle, flippers, flukes and dorsal fin. For one dolphin (Case 3), the periorbital skin was so affected that the eyes were obscured. For many of the lesions, presumably more chronic, there was a thickened rough or velvety surface crust or mat that was often green, yellow or orange due to growth of fungi and/or algae.

### Histopathology

**Figure 5:**
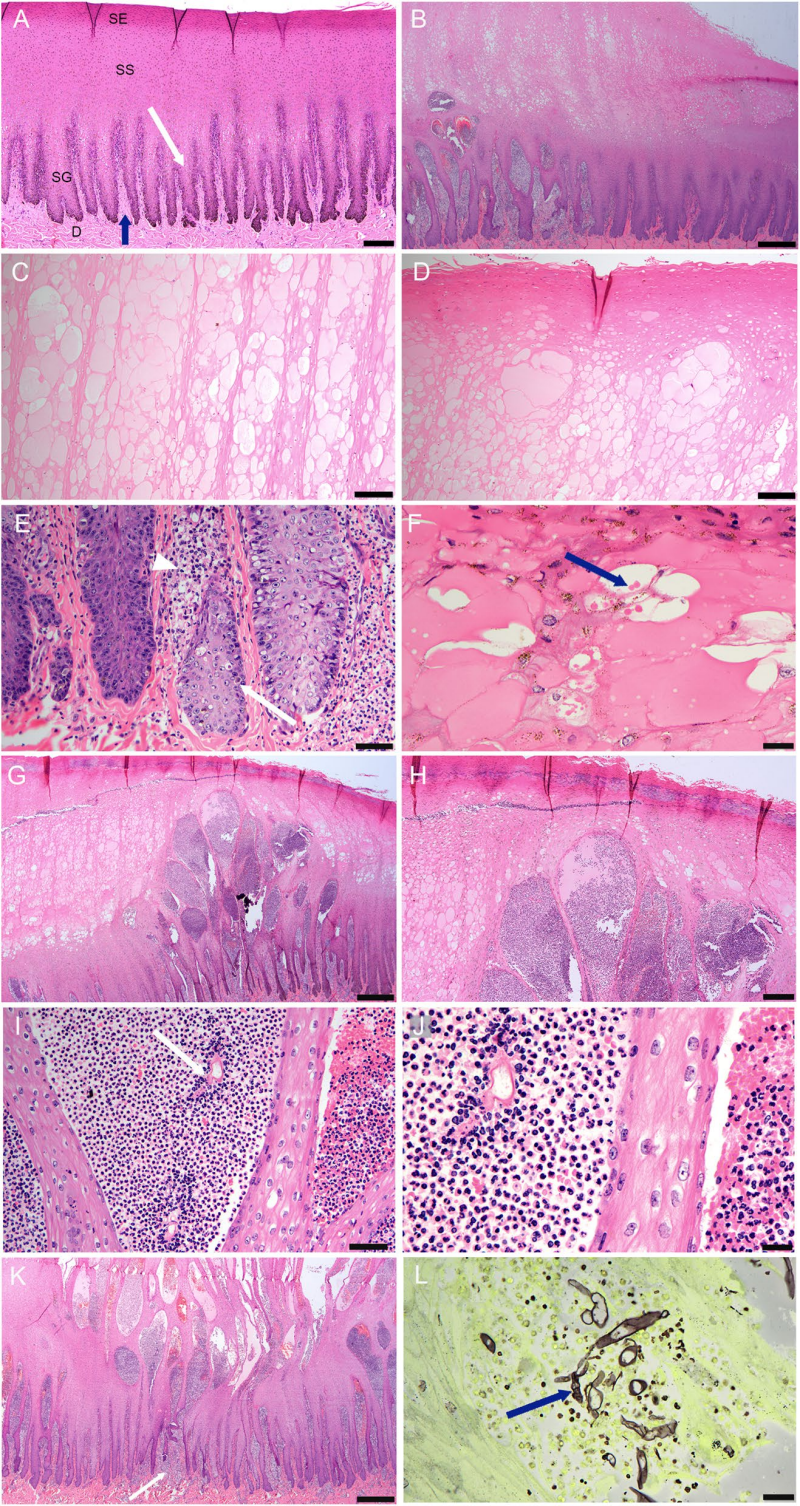
Histopathology of skin from *T. australis* and *T. aduncus*. Tissues are stained with hematoxylin and eosin unless otherwise stated. (**A**) Normal skin from a bottlenose dolphin (*T. aduncus*) showing the *stratum externum* (*SE*), the *stratum spinosum* (*SS*) with acanthocytes arranged in vertical columns, the *stratum germinativum* (*SG*). The surface area of the latter is greatly expanded by the epidermal papillae (white arrow) extending down and the dermal papillae (blue arrow) extending up from the dermis (**D**). Bar = 200 µm. (**B**) *T. australis,* Case 1*,* skin showing an abrupt transition (right to left) from relatively normal epidermis to marked acanthosis at the periphery of a large ulcer (*cf* 2D above). The acanthocytes are hypertrophied and pale (intracellular edema, hydropic change). Dermal papillae to the left are also expanded. Bar = 500 µm. (**C**) *T. australis,* Case 1*,* skin, *SS* mid zone, with marked hydropic change and early vesicle formation in multiple acanthocyte columns. Bar = 100 µm. (**D**) *T. australis,* Case 1*,* skin at *SE/SS* interface showing the formation of vesicles immediately below the *SE* characterized by hypereosinophilia and nuclear pyknosis (coagulation necrosis). Bar = 100 µm. (**E**) *T. australis,* Case 2*,* skin at the epidermal-dermal interface showing pallor (intracellular edema and vacuolation) of basal acanthocytes in the epidermal papillae to the right relative to the more normal cells in papillae to the left. There are also increased apoptotic cells in the affected papillae (arrow). Infiltration and extravasation of neutrophils with sub-epidermal edema is indicated (arrow head). Bar = 100 µm. (**F**) *T. australis,* Case 2*,* skin. *SS*, higher power view (× 400) of degenerating acanthocytes with numerous eosinophilic keratohyaline bodies (arrow). Bar = 20 µm. (**G**) *T. aduncus,* Case 4, skin. Expansion of a cluster of dermal papillae radiating towards the middle of the *SS.* The papillae are expanded by neutrophils, erythrocytes and fibrin (pustules). The *SS* is markedly expanded by severe diffuse hydropic change in acanthocytes. Bar = 500 µm. (**H**) *T. aduncus*, Case 4, skin. Higher power view of G (× 200) showing the pustules expanding into the superficial layers of the *SS* and the marked hydropic change in adjacent acanthocytes. Bar = 200 µm. (**I**) *T. aduncus*, Case 4, skin. Higher power view (× 200) of adjacent dermal papillary pustules showing degenerating neutrophils admixed with fibrin (arrow) and erythrocytes admixed with leukocytes. Bar = 50 µm. (**J**) *T. aduncus*, Case 4, skin. Higher power view (× 400) of (I) showing cellular composition of pustules. The epidermal papilla between pustules has mild hydropic degeneration. Bar = 20 µm. (**K**) *T. aduncus*, Case 4, skin. Low power view (× 20) showing a series of dermal papillary pustule clusters arising along the epidermal-dermal junction. Centrally there is more marked focal superficial dermatitis (arrow) and incipient ulceration to the surface through the overlying epidermis. Bar = 500 µm. (**L**) *T. aduncus*, Case 4, skin. Epidermal surface crust showing a mixed growth of fungal hyphae (arrow), Grocott-Gomori’s methenamine silver stain. Bar = 20 µm.

For reference, normal bottlenose dolphin skin is shown (Fig. 5A). Skin sections from areas grossly described as pale, were characterized histologically by increased depth of the stratum spinosum due to hydropic swelling (ballooning degeneration) of acanthocytes (Fig. 5B). There was often an abrupt transition between relatively normal epidermis and the areas of hydropic change and epidermal swelling that increased the epidermal depth by up to twice normal (Fig. 5B). The stratum externum (equivalent to stratum corneum) was usually intact and there was no inflammation. As cell swelling progressed, acanthocytes became markedly distended with pale eosinophilic cytoplasm leading to cell rupture and the formation of intraepithelial vesicles (Fig. 5C,D). Hydropic change segmentally extended to the deeper cells of the stratum spinosum (Fig. 5E). Degenerating acanthocytes often had prominent eosinophilic cytoplasmic bodies characteristic of keratohyaline granules and tonofibrils (Fig. 5F). Occasionally these resembled poxviral eosinophilic intracytoplasmic inclusion bodies. The increased depth and focal swelling of the stratum spinosum distended the overlying stratum externum resulting in breaches and erosion of this layer and coagulative necrosis characterized by hypereosinophilia and pyknosis of acanthocyte nuclei (Fig. 5D). The eroded surface frequently was capped by accumulated cellular debris and was infiltrated by morphologically diverse hypha-like elements, bacteria and diatoms. The deeper stratum spinosum was usually less affected and the germinal layer was generally unaffected. Changes in the superficial dermis and dermal papillae were mild and usually focal characterized by congestion of the vessels, endothelial hypertrophy, perivascular edema and extravasation of neutrophils, all of which expanded the papillae (Fig. 5E).

Progression of the lesion from the exterior resulted in full depth loss of the epidermis and ulceration as the basement lamina was breached. However, in many of the sections examined, the lesion appeared to progress from the inside out with marked neutrophilic infiltration from the dermal papillae infiltrating into the degenerating stratum spinosum and filling the vesicular spaces creating multifocal to coalescing intra-epidermal pustules filled with degenerating neutrophils and fibrin (Fig. 5G–J). These eventually erupted through the stratum externum as the exudative ulcers described grossly (Fig. 5K). In the dermis and superficial hypodermis, mild edema and occasionally focal hemorrhage with variable leukocytic infiltration was sometimes noted. Dermal papillary arterioles and superficial dermal arterioles occasionally had subtle hyper-eosinophilia of the tunica media, suggestive of fibrinoid necrosis. In the stratum externum and superficial necrotic crust, numerous non-pigmented mycelial fungi with broad hyphae that varied in size and width, often with nonparallel walls, irregular right-angled branching, and rare septations and budding were present. They typically stained poorly with PAS but were more obvious using Grocott-Gomori, Gram or Giemsa staining methods (Fig. 5L). In some areas, hyphae extended to the dermo-epidermal junction and rarely, into the superficial dermis. Usually trapped within the surface crust were mixed bacterial colonies, algae with thick cell walls and other cellular and acellular debris.

The morphologic diagnosis generally included the descriptors: Multifocal, vesiculo-pustular and ulcerative dermatitis with hydropic degeneration of the stratum spinosum and secondary bacterial and fungal infection. The temporal descriptors were generally acute to subacute, as granulation tissue was not observed in the samples collected from cases described. However, as skin samples were generally collected at the margins of lesions rather than centrally, granulation tissue may not have been sampled.

### Ancillary testing

Skin samples were cultured for bacteria and fungi. Isolates were mixed and inconsistent between cases and included several gram-negative bacteria such as *Vibrio spp*. (unspeciated), *V. anguillarum*, *V. alginolyticus, V. vulnificus* and *Shewanella putrefacsciens*. Fungi included *Saprolegnia* spp., *Candida* spp., mixed non-pathogenic fungi (species not specified) and PCR for *Aphanomyces invadans* was negative. The eosinophilic inclusion-like bodies in acanthocytes were negative for pox and herpes virus conserved sequences by PCR using published primers^44,45^. Furthermore, no pox- or herpesviral particles were detected on transmission electron microscopy, with the eosinophilic inclusion-like bodies in acanthocytes instead confirmed to be composed of aggregates of desmosome components (Australian Animal Health Laboratories, Geelong, Victoria).

### Environmental factors

#### Gippsland lakes

The Environmental Protection Agency for the State of Victoria had a water monitoring program in place prior to the October–November 2007 mortality event in the Gippsland Lakes^46^. The program focused on monitoring a range of ecologically relevant water quality parameters hourly using Hydrolab Sondes DS5X (automated water quality instruments) fitted with sensors for pH, salinity (ppt), temperature (C), depth (m), dissolved oxygen (% saturation) and chlorophyll-a (µg/L). The monitors were located 50 cm beneath the water surface, and in water depth greater than 5 m, a second monitor was located 50 cm above the bottom. Eight sites were selected in Lakes King and Victoria, the two lakes closest to the town of Lakes Entrance where the lakes connect by an engineered channel to the Tasman Sea. As such, these bodies of water have the most marine environment compared to the other lakes that are closer to the fresh water riverine inflow.

**Figure 6:**
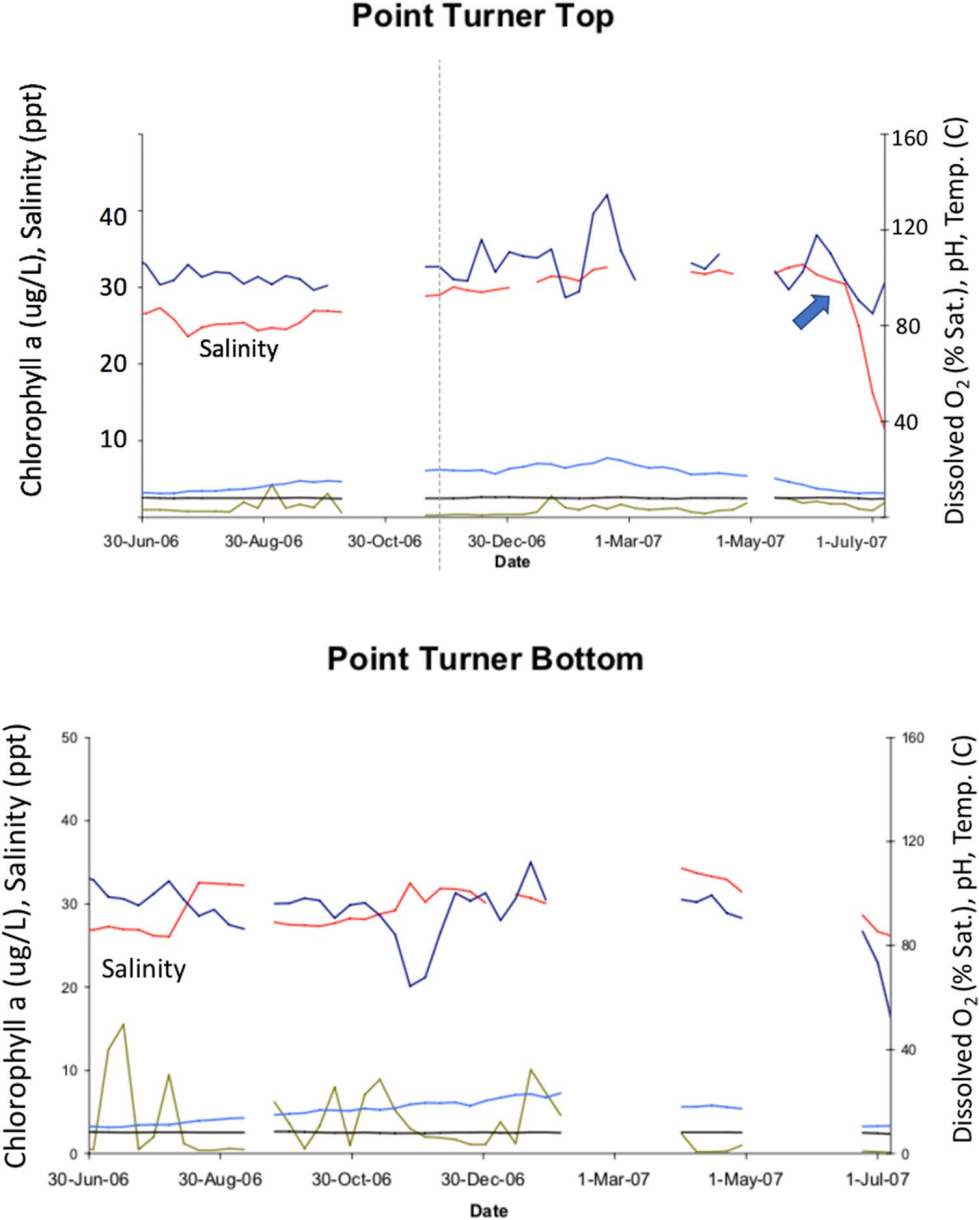
Physical and chemical properties at Turner Point, Lake King North, Gippsland Lakes, Victoria, for 2006–’07. The red line shows surface salinity (ppt) declining rapidly and markedly from late June through early July 2007 (arrow, upper graph) with formation of a halocline as the bottom salinity is only slightly decreased (lower graph). Surface temperature (C, light blue line), dissolved oxygen (% saturation, dark blue line), chlorophyll a (µg/L, green line) and pH (black line) are also depicted. Source^46^.

**Figure 7:**
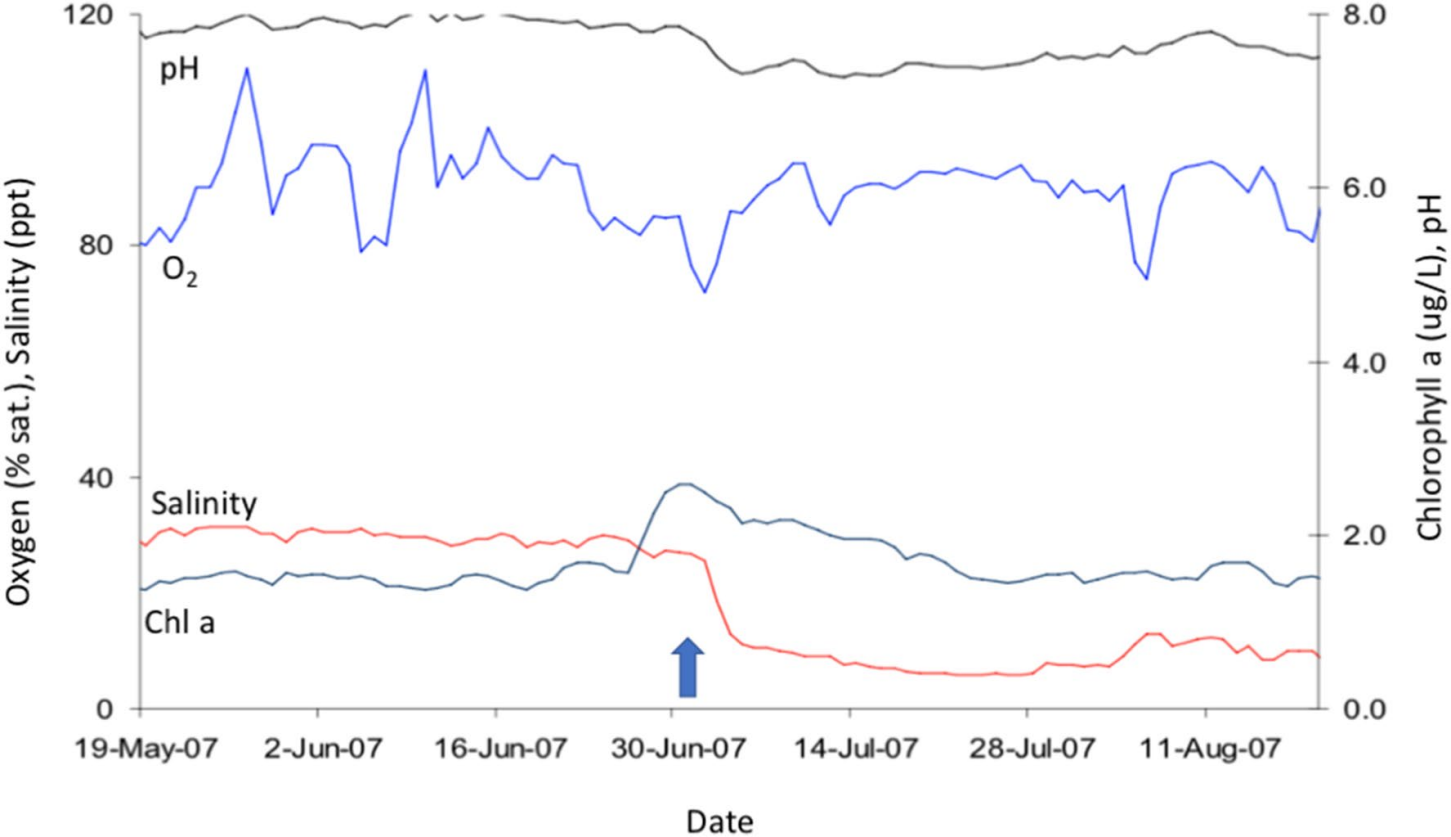
Water quality at McLennan’s Strait, Lake Victoria, showing the rapid decline in salinity (red line and arrow) in late June and sustained levels at 10 ppt or less through the period of monitoring at the end of August, 2007. Source^46^.

Data from the monitoring stations showed that levels of salinity varied during the course of the year but were generally between 25 and 35 ppt^46^. Analysis of historic EPA data (1986–1996) fixed-sites network indicated that the median surface water salinity in Lake Victoria was 16.2 ppt and Lake King 19.0 ppt, with benthic waters having median values of 21.9 ppt and 31.7 ppt. The 2006–07 salinity levels were attributed to low annual rainfall resulting in stronger influence from marine (saline) inputs compared to river (freshwater) inputs^46^. From late June to early July 2007, the Gippsland Lakes were subjected to heavy sustained rainfall resulting in a “hundred-year” flood event. From late June through 1^st^ July, surface salinity at two stations on Lake Victoria and one on Lake King salinity declined rapidly from > 30 ppt and > 35 ppt respectively, to between 5 and 10 ppt (Figs. 6 and 7)^46^. In November another large flood, affecting the rivers located in the western part of the lakes system (Lakes Latrobe, Thomson, Avon) and the Mitchell River, added more fresh water, nutrients and sediments to the dolphin habitat, resulting in widespread changes in the water quality including decreased salinity. A large bloom of the freshwater cyanobacterium *Synechoccus* sp. developed in December 2007 that lasted for most of 2008^47^.

At deep water sites close to Lakes Entrance, a clear halocline was established during the June 2007 floods. For Lake Victoria, the surface salinity on 1^st^ July 2007 was only 12 ppt but at the bottom (approx. 9 m) it was 28 ppt. Stratification due to haloclines and thermoclines reduced mixing between benthic and surface waters and resulted in anoxic conditions at depth, due to consumption of oxygen by organisms metabolizing nutrients in the benthic waters. Surface water temperature from 20th July through 26th November ranged from 12 to 19.9 °C^46^.

#### Swan-Canning river

The SRT, now subsumed into The Department of Biodiversity, Conservation and Attractions (DBCA), Government of Western Australia, conducts weekly monitoring of water physical and chemical characteristics using an array of 19 fixed stations along the Swan River and eight along the Canning River. The stations record salinity (ppt), temperature (C), dissolved oxygen (% saturation) and chlorophyll-a (µg/L) from surface to substrate and weekly plots are published (https://www.dbca.wa.gov.au/science/riverpark-monitoring).

**Figure 8:**
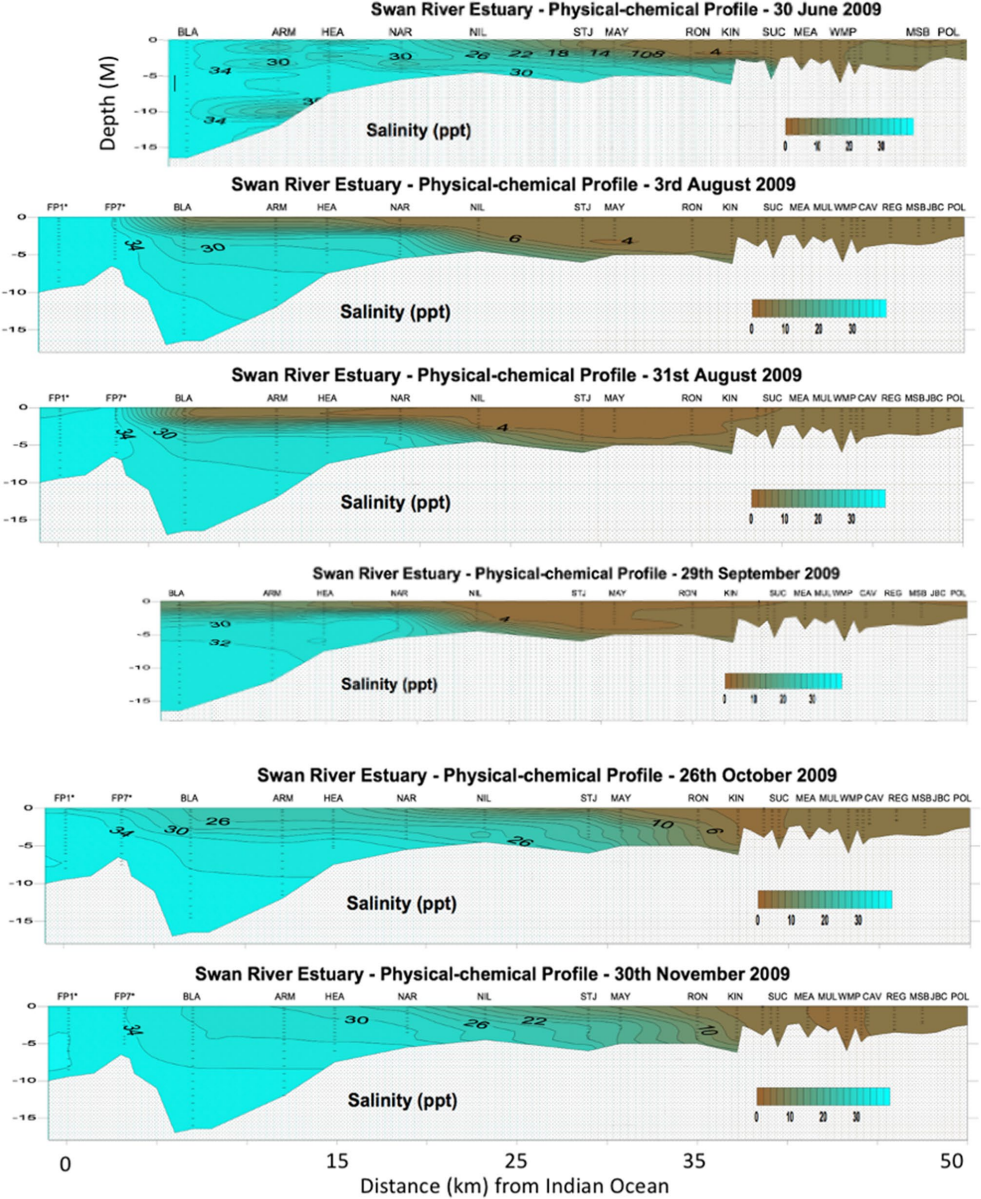
Temporal series of water salinity plots for up to 20 sampling stations (X axis top) at increasing distance from the Indian Ocean (0 on lower X axis) to 50 km upstream (right of figure). For reference to Fig. 1b, NAR is the Narrows Bridge. Fresh water (< 5 ppt) is represented as brown colors while marine water (> 25 ppt) is blue or green. Note that the surface water (up to 5 m depth) is predominantly fresh through the system from 3rd Aug. through 29th Sept. 2009. Prepared by the Department of Water and Environmental Regulation for the Department of Biodiversity, Conservation and Attractions, Western Australia.

**Figure 9:**
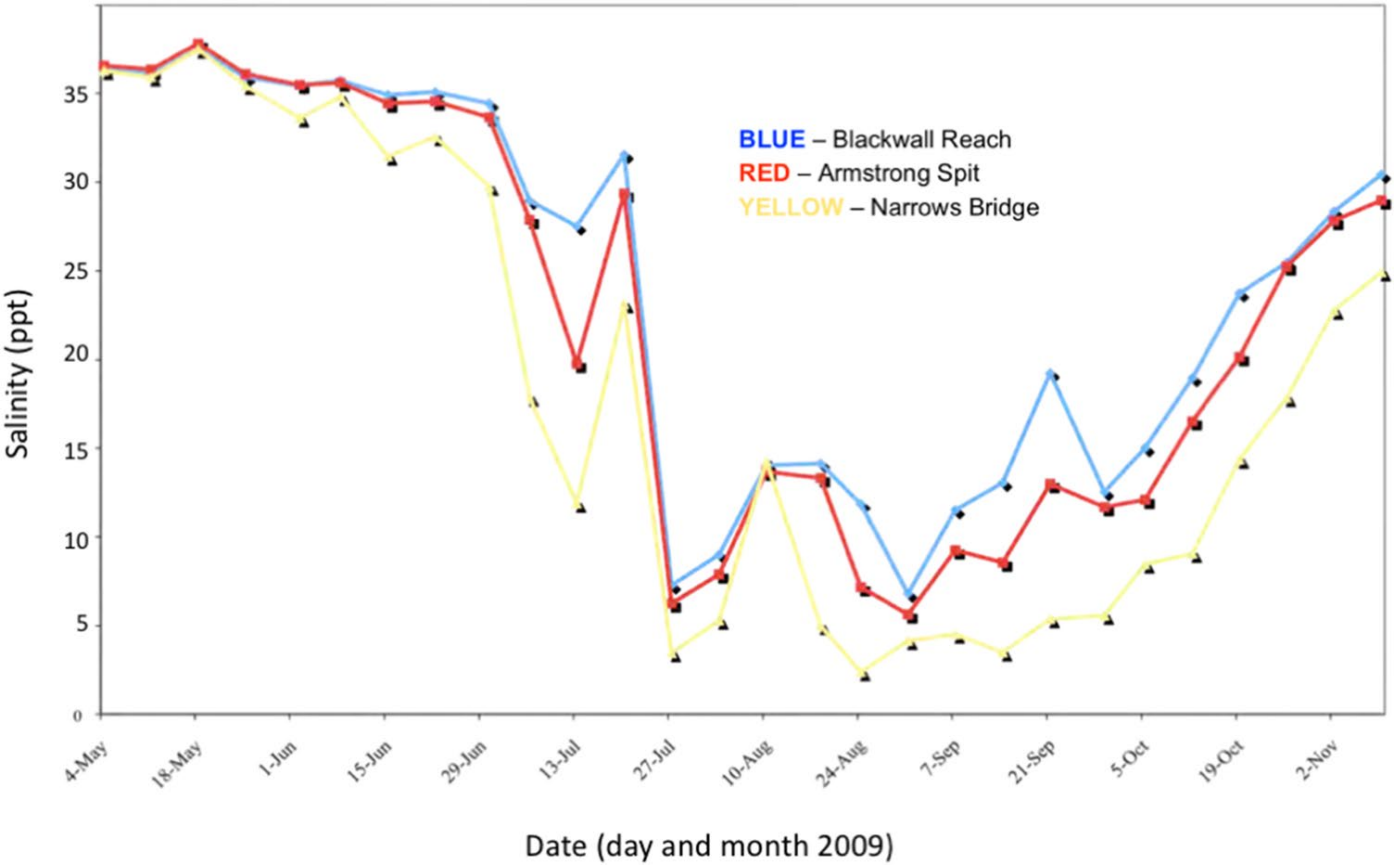
Surface (0.5 M depth) salinity of the Swan-Canning Riverpark at three locations from 4th May 2009 through 2nd Nov. 2009. Salinity fell rapidly in July to 5 ppt or less and remained below 15 ppt through early or mid October. Prepared by the Department of Water and Environmental Regulation for the Department of Biodiversity, Conservation and Attractions, Western Australia.

From late June through July 2009 there was heavy rainfall in the Swan and Canning catchments that resulted in rapid runoff to the rivers and a decrease in salinity over an extensive length of the river systems (~ 50 km). The influence of freshwater flow switched the Swan River from a predominantly marine system (25–35 ppt) to brackish (5–25 ppt) and ultimately, fresh-water (< 5 ppt) from July through October when the system reverted to a more marine environment (Fig. 8). Weekly water quality sampling at the Narrows Bridge south of Perth, indicated that surface salinities at that location were < 15 ppt from 3rd July 2009 through19th October, and for seven weeks of that time ranged between 2 and 5 ppt (Fig. 9). The degree to which this change was observed and the period over which it occurred was dependent on site and the relative influence of both freshwater flow vs. tidal influence, with more upstream sites experiencing lower salinities for longer periods than downstream sites (Fig. 8). As with the Gippsland Lakes, a halocline was present throughout much of this period at the lower reaches of the system. Equivalent rapid salinity changes also occurred in the Canning River^48^. The Kent Street weir on the Canning River was opened in late June and the saline conditions of the river downstream of the weir rapidly changed to fresh and remained that way through October 2009. Surface water temperatures throughout much of this period ranged from 17 to 21 °C ^48^.

## Discussion

It is unknown how cetaceans sense water salinity but it appears that the one sense of taste that they retained through evolution is that for salt^49^. Field studies on common bottlenose dolphins that normally inhabit a transition zone between fresh and marine water supports the contention that they actively avoid prolonged contact with hypo-saline water^50^. Satellite telemetry of tagged dolphins in Barataria Bay, LA, found that they had a likely salinity threshold tolerance of ~ 8 ppt. They were most frequently found in waters with higher salinities and very rarely found in waters with salinities below ~ 8 ppt^50^. Clearly the lower salinity levels recorded for the two study sites here were below the preferred levels for bottlenose dolphins. Furthermore, the salinities were in the range of those where skin pathology and mortality had been reported for dolphins in Lake Pontchartrain, LA (~ 4.8 ppt) and the bays of East Texas (< 10 ppt) over extended periods^13,14,51^.

Early clinical records and research on captive dolphins noted that animals maintained in fresh water for days, or hypo-saline water for extended periods, were adversely affected. A consistent clinical manifestation was severe skin lesions that became progressively worse with prolonged exposure^52–54^. A recent analysis of medical records for 46 US Navy dolphins collected over 43 years, documented serum chemistry changes and dermatitis that varied inversely with the level of salinity to which the animals were exposed^55^. Similar skin changes and lesions were reported for cetaceans either in natural habitats subjected to a salinity change for various reasons, or for individual animals who ended up in fresh water either by misadventure or following unusual weather or some other catastrophic event^9,13,14,51^. In addition to dermatitis and secondary infection, there are serum electrolyte imbalances including decreased osmolality and sodium and chloride levels associated with over-hydration due to increased transcutaneous water absorption and solute loss^56–59^. Corneal opacity (edema) has also been observed in dolphins residing in hypo-saline waters for prolonged periods or out-of-habitat dolphins rescued and repatriated from freshwater systems^51,59,60^. The associated physiological stress of prolonged freshwater exposure eventually leads to mortality, as for the four dolphins in this report, but the pathophysiology is not completely understood. However, it is likely to include severe electrolyte imbalances, renal and endocrine decompensation, cerebral edema, and probably terminal endotoxemia/septicaemia^4,60–63^. While the gross appearance of skin lesions has been described from photographs of free-living dolphins^13,14,51^, there are no published reports on the dermatopathology of such cases.

The goal of this study was to address this issue by describing the gross and histopathologic skin lesions in free-ranging bottlenose dolphins with a known history of sudden, profound and prolonged exposure to hypo-saline water. In order to achieve this goal, a number of elements had to align including an understanding of the ecology of the dolphin communities prior to the event characterized by ulcerative dermatitis; the environmental characteristics of the habitat (physical and chemical profiles recorded at high frequency preferably over many years); ideally the identity and residency patterns for individual animals; retrieval of carcasses in a timely manner and necropsy by an experienced pathologist; and continued monitoring of survivors and of the environment. For other suspected outbreaks of freshwater skin disease, some of these elements, particularl retrieval of fresh carcasses for necropsy, were missing e.g.^13,14,51,59,60^.

In this study, both ecosystems have known resident dolphin populations that had been the subject of ecological and behavioral studies for many years^18–20,41^. In addition to specific scientific studies on dolphin ecology in the Swan-Canning Riverpark, since the 2009 mortalities, a citizen science initiative was started to monitor dolphins in the Riverpark. Dolphin Watch is a partnership project between the Swan River Trust’s River Guardians Program, Murdoch University, and Edith Cowan University (Curtin University of Technology was formerly a partner). Since its inception in autumn 2009, more than 150 River Guardians volunteers have received training in dolphin observation and data recording techniques and collect data on the occurrence of dolphins throughout the Riverpark (see further at: http://ww.riverguardians.com)^48^. More recently, the Marine Mammal Foundation has instigated a similar citizen science program, Burrunan Watch, for Gippsland Lakes. Volunteer Lakes Champions monitor and record the occurrence of Burrunan dolphins, dolphin behaviour and vessel interactions and/or violations. For both locations, the previously established network of scientists and citizen-scientists enabled the timely recording of affected dolphins and retrieval of some of the mortalities for detailed examination.

**Figure 10:**
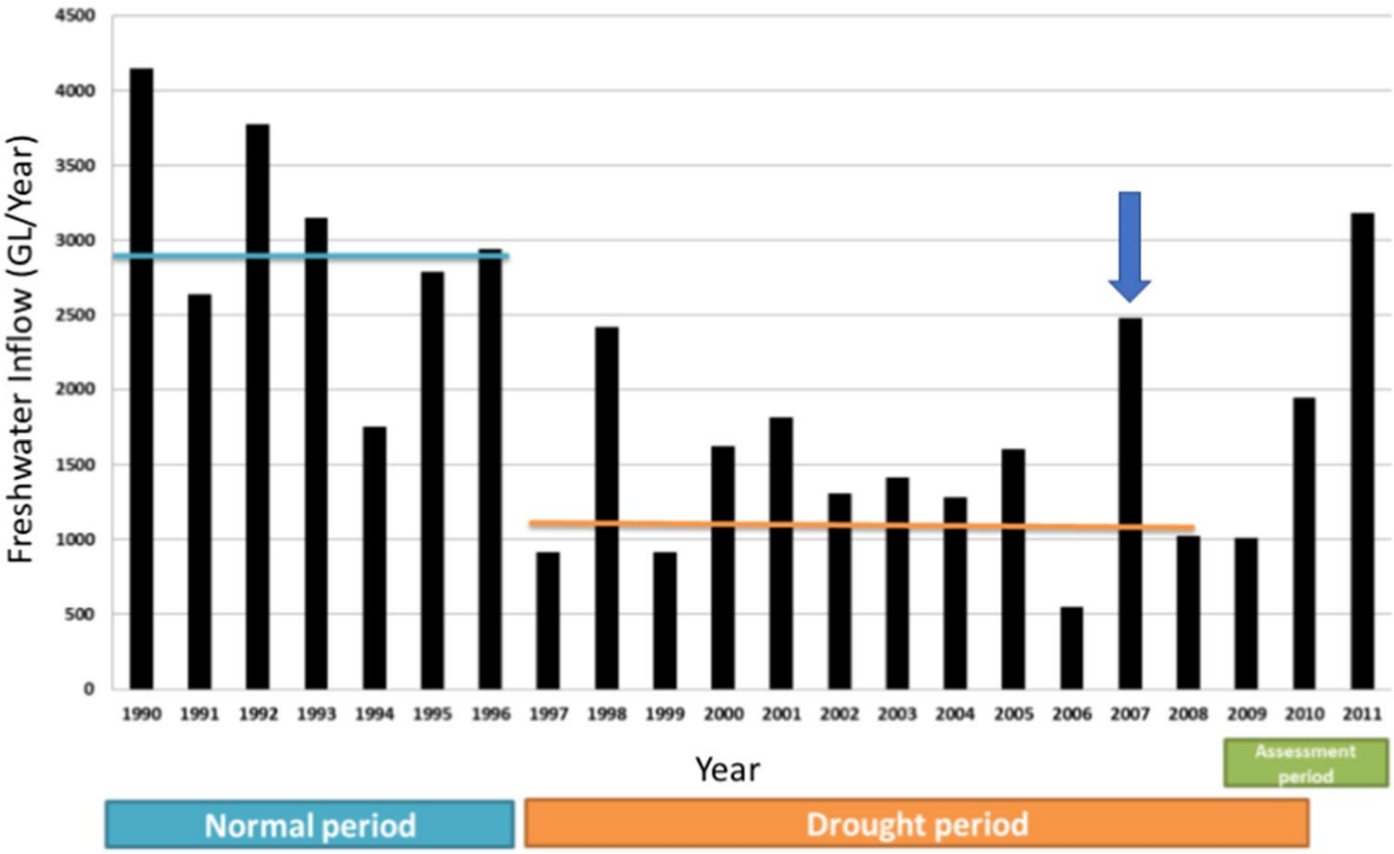
Freshwater inflow to the Gippsland Lakes, Vic., in gigalitres per year (GL/Yr) for 1990 to 2011 showing that the heavy rain of 2007 (arrow) represented an abrupt return to normal inflow after a 10 year period of drought. Source^46^.

**Figure 11:**
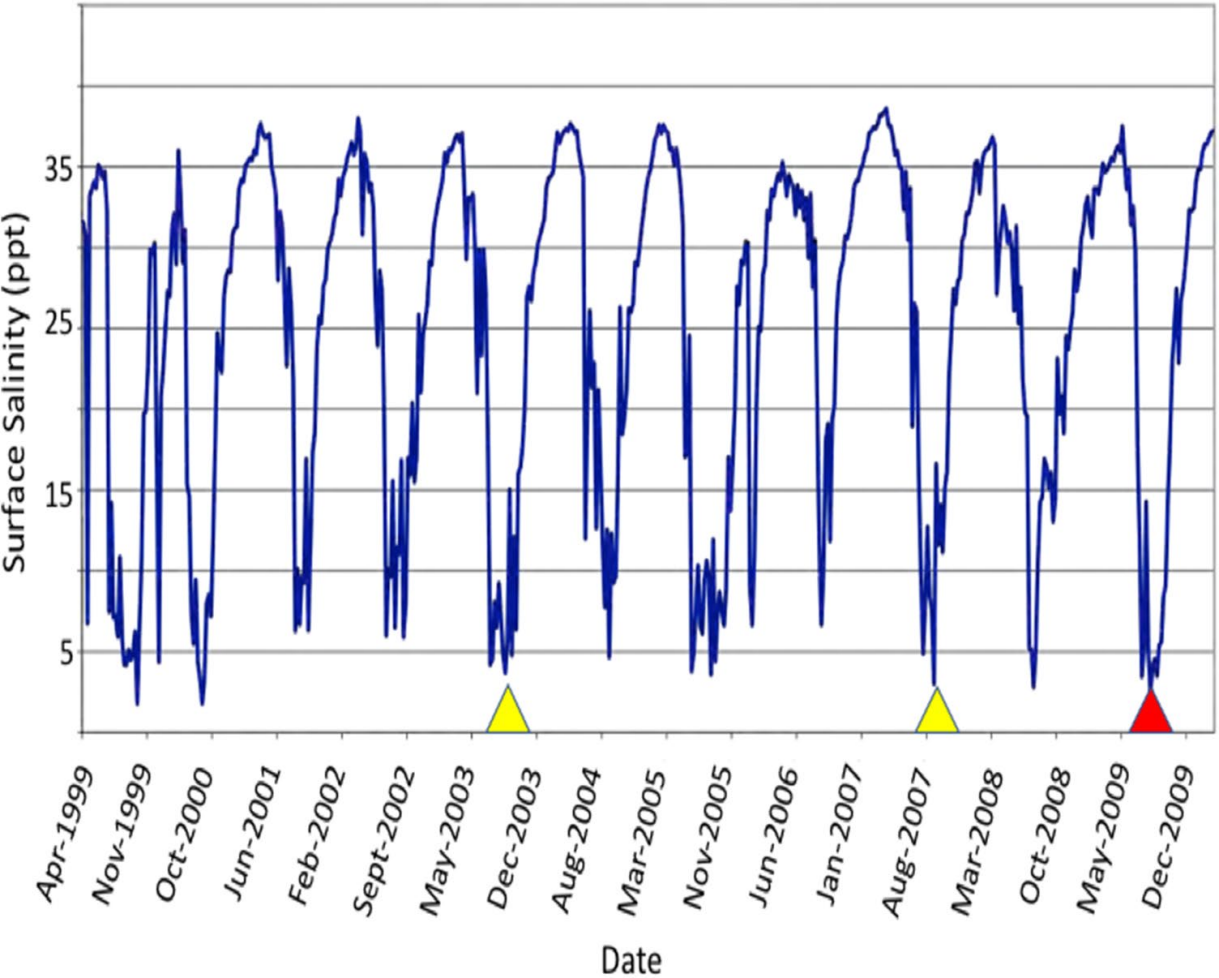
Surface (0.5 M depth) salinity at the Narrows Bridge in the Swan-Canning Riverpark from 1999 through 21st Dec. 2009 showing marked annual seasonal declines in salinity from late austral winter through spring. Confirmed (red arrowhead) and suspected (yellow) freshwater skin disease cases in resident bottlenose dolphins. Prepared by the Department of Water and Environmental Regulation for the Department of Biodiversity, Conservation and Attractions, Western Australia.

Environmental monitoring, and critically, the physical and chemical characteristics of the water in habitat frequented by dolphins, had been monitored at multiple sites, at variable depths, and over extended time periods at both study locations. For the Gippsland Lakes, the monitoring stations documented the sudden marked decline in salinity and other aspects of water quality first after heavy rain and flooding in June 2007 and again in November 2007, prior to, and during the onset of morbidity and mortality in the resident dolphins. In addition, long term monitoring showed that this event occurred at the end of a ten-year drought that began abruptly in 1996–97 and decreased freshwater inflow (salinity < 0.2 ppt) to the lakes by on average 53% (Fig. 10)^46^. As a consequence, the dolphins had been living in a system with annually increasing salinity (from mean ~ 15 ppt to 25 ppt) until the rains of 2007^64^ after which surface salinity decreased dramatically to 5 ppt or less and remained low for up to four months. Seasonal changes in salinity of the Swan and Canning rivers are not uncommon and lower salinity occurs annually every late winter to early spring (Fig. 11). There is, however, some inter-annual variation in the degree to which this occurs that is affected by rainfall in the catchment and tidal influence in the downstream reaches of the system. Salinities in the Swan Canning Riverpark between July and October 2009 were clearly low enough to be physiologically stressful and even pathologic to dolphins. Previous mortalities of Swan river dolphins with skin ulceration (October 2003, 18th November 2007) also coincided with relatively long periods (3 to 4 months) of marked hypo-salinity (Fig. 11). However, there have also been long periods of lowered salinity in other years (e.g. 2005) when no dolphin deaths were reported and in all years where dermatitis and mortality occurred, only a small number of animals appear to have died. There are probably many reasons for this but factors that influence exposure to low salinity for a prolonged period include individual behavior such as site fidelity, residency patterns, use of refugia (haloclines) where salinity levels are higher, and possibly, prey selection. At both locations, dolphins were free-ranging and had access to either the Indian Ocean or Tasman Sea and also to water of higher salinity beneath the halocline. Based on follow-up boat-based surveys in the Gippsland Lakes, there was clearly a wide variation in the severity of skin lesions with only 40% of survivors that could be photographed showing evidence of dermatitis suggesting that the majority avoided continued contact with fresh water. A similar temporal pattern in the onset, progression and eventual resolution of skin lesions was documented by photographic surveys in Galveston Bay, TX, before, during and after the fresh water influx caused by hurricane Harvey (14).

The gross appearance of skin lesions we describe has been observed previously in common bottlenose dolphins from lagoons or estuaries in the northern Gulf of Mexico^13,14,51^. However, in these reports it was not possible to sample fresh carcasses for histopathology. The lesions described here, suggest a pathogenesis implicating osmotic stress on the cells of the stratum spinosum with marked hypertrophy and expansion of the depth of this layer as the first observable change. At this stage, the gross circular or targetoid appearance of the lesions and the hydropic ballooning of acanthocytes and eosinophilic intra-cytoplasmic bodies can resemble cetacean pox^1,65–67^. Attempts to identify cetacean pox by PCR and TEM were unsuccessful which was not surprising given that it is a disease more common in juvenile dolphins and not adults as in this study^68–70^. Under conditions of chronic stress, social or environmental, pox outbreaks can occur and lesions may be present on older dolphins, particularly males^10,11,71,72^. However, while these lesions may be large, they retain their tattoo-like character and only rarely result in ulceration^73^. It could be argued that pox lesions existing prior to immersion in fresh water could act as a portal of entry for fresh water and exacerbate the progression of dermatitis. If that were the case, then we would have expected that juvenile animals would have been more affected during the described fresh water event rather than adults as recorded here.

Fresh water skin disease may result from periodic seasonal freshwater flushing of estuarine habitat (e.g. Swan-Canning River), unusual flooding of intracoastal waterways (e.g. Gippsland Lakes), or potentially degraded estuarine/coastal habitat subject to freshwater flooding (e.g. northern Gulf of Mexico^13,14^). In all of these scenarios while coastal bottlenose dolphins (*Tursiops* spp.) have been the focus species affected, other delphinids and baleen whales are potentially susceptible. In South America, Sanino et al.^74^ documented multifocal ulceration on the skin of an adult male Chilean dolphin (*Cephalorhynchus eutropia*) initially photographed alive but subsequently stranded on Tonina Beach in the Añhiué Reserve, Patagonia. The beach is at the mouth of the Palena River and the local waters are heavily influenced by fresh water inflow with surface salinity ranging from 15 to 20 ppt. At several coastal estuarine locations in Brazil, where surface salinity approaches fresh water, extensive green–brown and orange skin plaques have been described on free-ranging Guiana dolphins (*Sotalia guianensis*) but ulceration or mortalities were not reported^75^. Dermatitis consistent with fresh water skin disease was also reported for a humpback whale (*Megaptera novaengliae*) mother and calf that were entrapped in the Sacramento River in Northern California for a prolonged period^9^. It is worth noting that fresh water skin disease is a condition limited to cetaceans that evolved in a marine environment. The polyphyletic river dolphins from southern Asia and South America, evolved in riverine habitats, and lesions as described in this report, have not been reported^76–78^.

In conclusion, the consistency of lesion morphology and pathogenesis, clinical course and outcome of two events affecting coastal bottlenose dolphins in Australia permitted the development of a case definition that is presented here. The common elements included a sudden (within days) and marked (from > 25 ppt to < 5 ppt) fall in salinity, prolonged (weeks to months) exposure to hypo-saline conditions, development of skin lesions characterized by hydropic degeneration and epidermal expansion leading to vesicle formation and resulting in gross pallor, erosion and ulceration, often with intra-epidermal pustules and eruption that grossly appears as exudation, and secondary bacterial, fungal and algal infection or overgrowth that manifests as green, brown or orange mats or plaques. Depending on the severity of lesions, duration of exposure to adverse conditions, access to halocline refugia, or intercurrent disease (e.g. renal dysfunction), and ambient temperature, the outcome may be complete resolution or death. This case definition will facilitate a better understanding of a dermatopathy that will likely continue to emerge where coastal dolphin communities globally are exposed to sudden or unprecedented environmental change as a result of climatic change or perturbations and anthropogenic habitat degradation.

## Data Availability

All data relevant to this study are included in the manuscript. There are no additional data sets.
